# Characteristics of achalasia and detection of pulmonary complications: A comparison of findings in younger and elderly patients

**DOI:** 10.5339/qmj.2024.52

**Published:** 2024-12-24

**Authors:** Jelena Jankovic, Branislava Milenkovic, Aleksandar Simic, Nenad Ivanovic, Ognjan Skrobic

**Affiliations:** 1 Clinic for Pulmonology, University Clinical Center of Serbia, Belgrade, Serbia; 2 Medical Faculty, University of Belgrade, Belgrade, Serbia *Email: jjelena1984@gmail.com; 3 Clinic for Digestive Surgery, University Clinical Center of Serbia, Belgrade, Serbia

**Keywords:** Achalasia, subtype, elderly, lung complications

## Abstract

**Background:**

Achalasia is a rare esophageal motility disorder of unknown etiology, which leads to changes in the pressure and relaxation of the lower esophageal sphincter (LES), affecting peristalsis and contraction of the esophageal body. Dysphagia can impact individuals of any age, it is frequent in the elderly. Non-specific gastrointestinal symptoms are delayed and can give false diagnoses. The aim of this study is to compare clinical presentation and pulmonary complications in younger (Group I) and elderly patients (Group II).

**Methods:**

108 patients with achalasia were separated into two groups—young and elderly patients. Demographic, clinical, radiological and manometric data, smoking status, and symptom score were compared between these groups.

**Results:**

There was no significant difference in gender, duration of symptoms, body mass index (BMI), or diameter of esophagus between the two patient groups. There was a statistically significant difference between frequencies of comorbidity between groups (*p* = 0.009). Even though there were no differences in chest tomography scan (CT) findings and diameter, there were statistical differences in diffusion capacity (*p* = 0.041). Respiratory symptoms occurred in 38 (48.7%) patients in Group I and in 20 (66.7%) in Group II (*p* = 0.011). Cough was dominant in the younger group, but fatigue and chest pain were statistically significant and frequent in elderly group patients with achalasia. There was no significant difference in Eskardt symptom score (ESS), but there was the difference in the frequency of individual symptoms. Vomitus and regurgitation were statistically higher frequent in Group I, but dysphagia and weight loss in Group II. Subtype 1 was dominant in the younger group, and subtype 2 in a group with older patients.

**Conclusion:**

The younger achalasia population group was found to be associated with decreased diffusion capacity, type 1 achalasia, cough, and gastrointestinal symptoms such as vomitus and regurgitation. Geriatric status was found to be associated with frequent comorbidities, subtype 2, frequent respiratory symptoms, dysphagia, and weight loss. Our findings demonstrated an association between esophageal motility abnormalities and characteristics of geriatric population.

## 1. Background

Achalasia is a very rare esophageal motility disorder that affects 1–2 people per 100,000 people worldwide. Etiology is still unknown, and barium swallow, endoscopy, and manometry can confirm diagnosis.^1^ Using manometry based on Chicago classification version 4 from 2021, achalasia is categorized into three subtypes: subtype 1 is an end-stage phenotype, subtype 3 is a spastic type, and subtype 2 is the most common type.^2^ Achalasia characterized a non-relaxing lower esophageal sphincter (LES), weak or absent esophageal peristalsis leading to an outflow obstruction at LES, and esophageal dilatation. Because of that, patients have difficulty in swallowing, regurgitation, vomiting, and weight loss as dominant symptoms. Symptoms are progressive over many years and not specific only for achalasia.^3,4^ This disorder has significant diagnostic delay for up to 5 years and is associated with false diagnoses. It remains unclear whether the cause of misdiagnosis is atypical clinical presentations or inconclusive diagnostics.^5,6^ According to literature, about 20–50% of achalasia cases are initially misdiagnosed given a first diagnosis such as gastroesophageal reflux disease, hiatus hernia, eosinophilic esophagitis, psychiatric, neurologic, cardiologic, or thyroid diseases.^5,7^ The problem of misdiagnosis can be two-way. On the one hand, there is insufficient knowledge of rare diseases and untimely recognition of them, and treatment cannot start on time, which leads to the progression of the disease and poor outcomes. Awareness of rare diseases is important in the education of health workers and all populations.^6^ On the other hand, patients often minimize or completely deny their symptoms. Perhaps the reason is fear or the unknowing about some disease, given that it is a subjective feeling and what the threshold of tolerance is remains questionable.^8,9^ Smokers often attribute their cough to years of smoking, abdominal pain to fast food and poor diet, headaches to migraines and stress at work, and shortness of breath to fatigue at work. All these can be warning signs of certain diseases.^9,10^ A geriatric assessment of symptoms and worsening of disease can refer to a person’s mental health, dementia, or the existence of Alzheimer’s disease.^11^

According to literature data, achalasia is more common in patients between 30 and 60 years and has two peaks of incidence, one in the 3rd to 4th decades and the other after 60 years. The diagnosis is mostly made in adulthood, and less often in childhood.^12^ Elderly people have aberrant esophageal motility, decreased LES tone, and more frequent upper gastrointestinal symptoms, which are generally attributed to age-related diseases, medications, and structural and functional changes in esophageal motility. Reasons for achalasia and aberrant motility are a lower number of ganglion cells causing degenerative changes in the intramural myenteric plexus and reduced esophageal peristalsis.^13^ The symptoms are more common and significantly impair the quality of life. In nursing homes, dysphagia rates among senior patients can reach 70% of all gastrointestinal symptoms, whatever underlying disease.^13^ This motility disorder and its most common symptoms, such as dysphagia and regurgitation, can cause aspiration of undigested food and can lead to structural pulmonary complications and can cause hospital treatment of aspiration pneumonia, and that way detect achalasia. Risk factors for the development of structural pulmonary disorders and aspiration pneumonia are older age, male gender, dementia, neurological diseases, immobilization, and dysphagia.^14,15^

The aim of this study is to compare clinical presentation and pulmonary complications in younger and elderly patients (taking into account information from literature about two peaks of incidence) with achalasia.

## 2. Methods

This retrospective study included 108 patients (older than 18 years) with a diagnosis of achalasia, treated at the University Clinical Center of Serbia. The current study enrolled 56 males and 52 females. Diagnosis of achalasia was made by manometry, and patients are divided into 3 subtypes according to the esophageal manometry. Exclusion criteria were neurological conditions influencing esophageal motility and pseudo-achalasia because of esophageal carcinoma. Characteristics of patients, age, smoking status, comorbidities, and other characteristics were obtained from medical history. Interviewing individuals with achalasia about their upper gastrointestinal symptoms, we got data, and based on that Eskardt symptom score (ESS) is calculated.^16^ A chest X-ray radiography, chest tomography scan (CT), diffusion capacity of the lungs (DLCO), and diffusion coefficient (KCO) on the device for measuring the lung transfer factor for carbon monoxide were performed before surgery as preoperative preparation. Patients were divided into two groups according to the World Health Organization age criterion. Younger patients were selected for Group I (<65 years old) and older adults for Group II (>65 years old).^17^

The study was approved by the Faculty of Medicine, University of Belgrade, Serbia (Ethics Committee number: 602/4).

The numerical data were represented as mean ± SD, and categorical data were reported as numbers and percentages (%). Descriptive and analytical statistical methods were use in this study. Student’s t-test and χ^2^-test were used also. Analyses were performed in IBM SPSS Statistics for Windows, version 22.0 (Armonk, NY: IBM Corp., 2019). Statistically significant differences were considered if the p-values were <0.05.

## 3. Results

**Figure 1.**
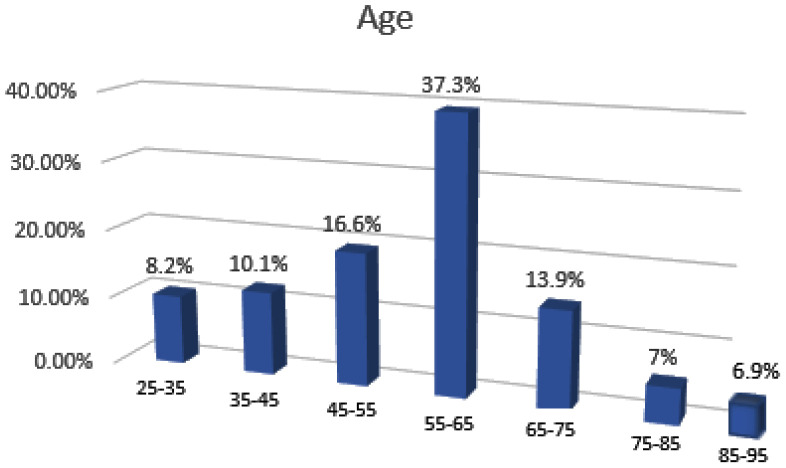
Distribution of patients according to age.

A total of 108 patients with achalasia were enrolled in the study. They were divided into the three achalasia subtypes according to the manometry and into two groups according to age (I and II). All of them completed the Eckardt questionnaires. The youngest patient had 25 years, and the oldest was 91. The distribution of patients according to age is presented in Figure 1. Of the 108 patients entering the study, 78 (72.2%) patients were in Group I, and 30 (27.8%) patients were in Group II.

Frequencies of specific characteristics of the patients between the two groups are shown in Table 1. There was no significant difference in gender, duration of symptoms, smokers, height, body mass index (BMI), or diameter of esophagus between the two patient groups. There was a statistically significant difference between frequencies of comorbidity between groups (*p* = 0.009). The most common were cardiovascular and obstructive (asthma and chronic obstructive pulmonary disease). All comorbid diseases are statistically frequent in elderly patients over 65 years. Even though there were no differences in CT findings and diameter, there were statistical differences in diffusion capacity (*p* = 0.041).

Subtype 1 was dominant in the younger group, and subtype 2 in a group with older patients. However, there were no differences in subtype 3 between groups.

**Figure 2.**
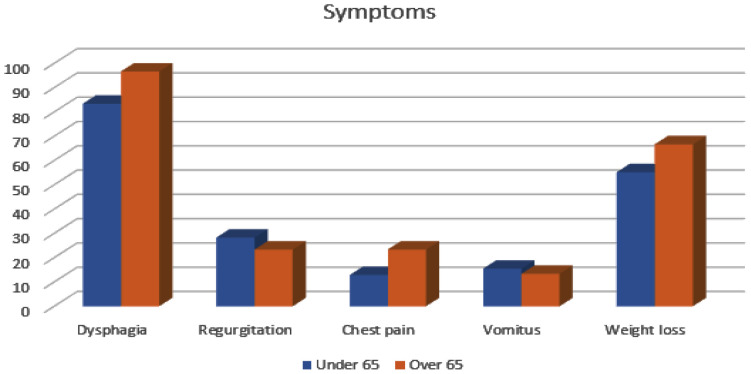
Symptoms frequency in different groups.

Respiratory and gastrointestinal symptoms and differences between groups are presented in Table 2. Respiratory symptoms such as excessive cough, chest pain, wheezing, dyspnea, and fatigue occurred in 38 (48.7%) patients in Group I and in 20 (66.7%) patients in Group II (*p* = 0.011). Cough was dominant in the younger group, but fatigue and chest pain were statistically significant and frequent in elderly group patients with achalasia. There was no significant difference in ESS, but there was the difference in the frequency of individual symptoms (Figure 2). Vomitus and regurgitation were statistically higher frequent in Group I, but dysphagia and weight loss in Group II. Weight loss was approximately 11 kg.

For all patients, treatment choice was laparoscopic Heller myotomy (LHM), and there were no complications. Patients were discharged after 2–5 days with good outcome.

## 4. Discussion

This study provides a clinical characterization of two groups of patients with achalasia. The youngest patient was 25 years old, and the oldest was nearly 91 years old. A third of patients are elderly. The most likely explanation for this frequency of geriatric patients is that achalasia is a chronic disorder with non-specific gastrointestinal symptoms that can persist for up to 30 years and have a low mortality rate.^18^ In most studies, the average age at establishment diagnosis was over the age of 50, which coincides with the highest number of patients in our study group precisely in the age group of 55–65 years.^19^ More than half of the younger group had manometry-certified subtype 1, and a similar percentage of the elderly group had subtype 2. This is not matched with a study by Roman and colleagues in which the age of the patients was similar between achalasia subtype 1 and subtype 2.^20^ But, the most recent study in Mexico, published in 2018, speaks in favor of our study. The results of that study showed that patients with achalasia subtype 1 were younger than those with achalasia subtype 2.^21^ Our possible explanation for that is in support of the theory of two peaks of incidence: in the younger group, the end stage of type 1 has already developed, while in the older ones, a later onset is possible, and the largest number just had subtype 2, and the terminal stage 1 did not develop yet. Also in support of this theory of two picks is the similar duration of symptoms in both of our investigated groups, which means that the existence of two peaks of the onset of the disease is still possible and at different times of beginning. There was no statistically significant difference in the age of the patients with achalasia type 3. That is consistent with literature data and also because this is the rarest type with an incidence of about 10%.^22^

As is well known, older patients have more comorbidities, but literature data showed that middle-aged patients and younger patients also have one or more comorbidities. Probably the reason is lifestyle and habits. The prevalence of multimorbidity increased substantially with age and was present in most people aged 65 years and older.^23^ Comorbidity leads to more difficult treatment, increased use of drugs, hospitalization, worsening of the underlying disease, and worse treatment outcomes, that seriously affect the quality of life of patients.^24^ It is worrying that in our study, in the group younger than 65, almost half of the patients had at least one associated disease. According to the data from previous studies, the most common comorbidity is cardiovascular disease (CVD) such as arterial hypertension, atrial fibrillation, and cardiomyopathy.^24^ Statistically, CVD and obstructive diseases were more common in the group of elderly patients (Group II) than is expected. In the younger population, predominant was asthma, and in the older group, it was chronic obstructive pulmonary disease.

In our study, we did not find differences in acute or chronic chest CT findings and diameter of the dilated esophagus, but there were minimal differences in diffusion capacity that were decreased in the younger group. The explanation for that may be in, as we previously said, the number of patients with end-stage subtype 1 in the younger group. In this subtype, peristalsis is absent, and undigested food is retained in the dilated esophagus and it is difficult to pass into the stomach. Because of that, symptoms such as cough as a consequence of aspiration after vomitus and regurgitation are most frequent. Frequent micro-aspiration indigested esophageal content can damage the alveolar-capillary membrane and lead to structural pulmonary changes such as fibrosis, nodular changes, pneumonia, or abscess.^14^ Damage of the alveolar-capillary membrane reduces diffusion through it, so the DLCO and KCO were decreased in a younger group with characteristics that are already mentioned.

Aging is characterized by progressive loss of physiological function, so gastrointestinal symptoms can also be a consequence of xerostomia, medication use, immobility, and neurologic conditions. That can lead to malnutrition, and unfortunately, most patients do not ask for a doctor’s help for years until the symptoms become very pronounced.^25^ Between our two groups, there was no difference in BMI and malnutrition status. Elderly subjects complained of dysphagia and weight loss more than patients in Group I. Dysphagia is a dominant gastrointestinal symptom in the geriatric population, who had or did not have proven achalasia with impaired motility and pressure at the level of the LES according to age.^26^ Regurgitation usually occurs in patients with almost complete esophageal dysfunction LES. It has been proven that approximately up to 38% of patients with achalasia have regurgitation and feel severe heartburn as a consequence of the fermentation of undigested food in the distal dilated esophagus.^27^ Our results are from the literature data and are predominant in the younger population in Group I. That can be explained by the majority of subtype 1 in the younger group with end-stage achalasia. Also, vomitus as frequent symptom in the elderly group is because the same reason. All of that causes cough as the dominant respiratory symptom in this group. Undigested food after regurgitation or vomiting can irritate oropharyngeal and bronchial mucosa and lead to irritating unproductive night cough.^27^ Weight loss was predominant in the elderly group, maybe because of dysphagia and fear of food intake and worsening of symptoms. Although pain is common in the group of older patients, there is an inadequacy in the assessment of retrosternal pain due to the presence of CVD and the correlation with the degree of achalasia is not strong, but it exists. Our results are in accordance with data from a study by Schechter and colleagues who concluded that younger patients had a higher prevalence of heartburn and chest pain than the elderly.^13^ Because of the higher prevalence of chest pain and fatigue, respiratory symptoms were significantly dominant in older people, even cough was dominant symptom overall.

For all patients, treatment choice was LHM, and there were no complications. LHM is a very successful method of treatment for patients with achalasia in any subtype, according to a study by Crespin and colleagues.^28^ Patients were discharged and recovered with good outcomes. This short period of hospitalization coincides with literature data.^29^

There are several limitations in this study. It is a single-center study, but the number is representative of other literature data because it is a rare disease. The second limitation is the small number of subtype 3 patients and the small number of patients in Group II, 2.5 times smaller than in Group I. We do not have an exact reason for no differences in the duration of symptoms between groups. A certain percentage of patients were not motivated to perform multi-detected computed tomography (MDCT), and they were excluded from study.

## 5. Conclusion

Achalasia is a prevalent cause of dysphagia in elderly patients. When older patients present with dysphagia, esophageal manometry can yield a diagnosis of achalasia. LHM is a safe and effective treatment even in elderly patients. The younger achalasia population group was found to be associated with decreased diffusion capacity, type 1 achalasia, cough, and gastrointestinal symptoms such as vomitus and regurgitation. Geriatric status was found to be associated with frequent comorbidities, subtype 2, frequent respiratory symptoms, dysphagia, and weight loss. In addition, our findings demonstrated an association between esophageal motility abnormalities and characteristics of the geriatric population.

## Acknowledgments

All authors have contributed equally towards the conception of the manuscript.

## Conflict of Interest Statement

The authors of this study declared that they have no conflict of interest.

## Authors’ Contributions

All authors contributed to the study conception and design. Data collection and analyses were performed by JJ and NI. The first draft of the manuscript was written by JJ, and all authors commented on previous versions of the manuscript. Critical revision and editing of manuscript: BM, AS, and OS. Data and statistical analyses: NI. All authors read and approved the final manuscript.

## Figures and Tables

**Table 1.** Frequencies of specific characteristics of the patients between two groups.

Group	I	II	p
N (%)	78 (72.2)	30 (27.8)	0.012
Gender			
*female	38 (48.7)	14 (46.7)	
*male	40 (51.3)	16 (53.3)	
Smokers	48 (61.5)	18 (60)	
Comorbidities			
*more than 1	37 (47.4)	26 (86.7)	0.009
*CVD	11 (14.1)	24 (80)	0.001
*obstructive disease	4 (5.1)	7 (23.3)	0.01
*Hypothyroidism	2 (2.6)	2 (6.7)	0.023
Duration of symptoms (years)	5.3	5.2	
Diameter (cm)	5.28	5.6	
CT pathological	40 (51.3)	15 (50)	
BMI	23.6	23.7	
Height (cm)	172.6	163.4	
Decreased diffusion capacity	24 (30.8)	8 (26.7)	0.05
Subtype 1	41 (52.6)	11 (36.7)	0.017
Subtype 2	29 (37.2)	16 (53.3)	
Subtype 3	8 (10.2)	3 (10)	

**Table 2.** Symptoms characteristics and frequency in different groups and comparisons among them.

Group N (%)	I	II	p
Respiratory symptoms	38 (48.7)	20 (66.7)	0.011
Cough	27 (34.6)	6 (20)	0.032
Chest pain	10 (12.8)	7 (23.3)	0.023
Fatigue	4 (5.1)	8 (26.6)	0.018
ESS	9.43	9.3	
Dysphagia	65 (83.3)	29 (96.6)	
Regurgitation	22 (28.2)	7 (23.3)	0.043
Vomitus	12 (15.4)	4 (13.3)	
Weight loss	43 (55.1)	20 (66.6)	0.05
